# Ethanol extract of *Lophatheri Herba* exhibits anti-cancer activity in human cancer cells by suppression of metastatic and angiogenic potential

**DOI:** 10.1038/srep36277

**Published:** 2016-11-03

**Authors:** Aeyung Kim, Minju Im, Min Jung Gu, Jin Yeul Ma

**Affiliations:** 1 Korean Medicine (KM) Application Center, Korea Institute of Oriental Medicine (KIOM), 70 Chumdan-ro, Dong-gu, Daegu 701-300, Republic of Korea

## Abstract

*Lophatheri Herba* (LH), dried leaf of *Lophatherum gracile* Brongn, has long been used to reduce thirst and treat fever and inflammation in Chinese medicine. Recent studies have shown that LH has anti-viral, anti-bacterial, anti-cancer, anti-oxidant, diuretic, and hyperglycemic properties. However, the effects of an ethanol extract of *L. herba* (ELH), at non-cytotoxic doses, on the metastatic and angiogenic abilities of malignant tumor cells have not been reported. We found that ELH significantly suppressed p38, JNK, and NF-κB activation and proteolytic activities under phorbol 12-myristate 13-acetate (PMA) stimulation, thus leading to a decrease in metastatic potential, including migration and invasion. In addition, ELH suppressed tumor-induced angiogenesis, including migration and tube formation in human umbilical vein endothelial cells (HUVECs) and microvessel sprouting from aortic rings via decreasing the pro-angiogenic factors in tumors. Interestingly, *in ovo* xenografts ELH-treated HT1080 cells did not increase in volume and eventually disappeared, owing to a lack of angiogenesis. Daily oral administration of ELH at 50 and 100 mg/kg markedly inhibited metastatic colonization of B16F10 cells in the lungs of C57BL/6J mice and caused no apparent side effects. These data collectively indicate that ELH is safe and may be useful for managing metastasis and growth of malignant cancers.

Malignant tumor cells exhibit key hallmarks, including uncontrolled growth, invasion into the surrounding extracellular matrix (ECM), metastatic spread, and angiogenesis; these features account for as much as 90% of cancer-related mortality^1^,^2^,^3^. During metastatic dissemination, cells in a primary tumor mass detach from ECMs, invade surrounding tissues, enter the blood and lymphatic vessels, survive and migrate through the bloodstream, exit and penetrate into distant new tissues, and finally adapt to the microenvironment and proliferate to form a secondary tumor^4^,^5^. In particular, matrix metalloproteinases (MMPs), the zinc-dependent endopeptidases known as matrixins, play an essential role in a wide range of tumorigenic processes, including tumor growth; metastasis via proteolytic degradation of ECMs such as collagens, gelatin, elastin, and proteoglycan; migration; and angiogenesis^6^,^7^. Numerous studies have demonstrated that the higher the expression of MMPs in tumors, the more aggressive the cancer. In addition, MMP levels correlate with poor tumor prognosis and relapse, thus suggesting that MMPs are possible predictors of tumor stage, metastasis, and recurrence^8^,^9^,^10^.

Angiogenesis is the sprouting of new vessels from pre-existing ones and is a normal physiological process in embryogenesis and development. In contrast, pathological angiogenesis is observed in several diseases, including rheumatoid arthritis, atherosclerosis, diabetic retinopathy, psoriasis, and cancer^11^. An extensive network of capillaries is necessary for continued tumor growth by providing sufficient nutrients and oxygen. In addition, tumor blood vessels act as paths for tumor cells to metastasize to distant organs from primary tumor foci. Tumors bearing higher vasculature have increased metastatic potential and subsequently become more aggressive tumors^12^,^13^. Folkman and colleagues have demonstrated that solid tumors cannot grow beyond 1–2 mm diameter without their own blood supply^14^. Angiogenesis occurs through a multi-step process that includes the release of pro-angiogenic factors, the release of proteolytic enzymes, the migration of endothelial cells (ECs) toward tumors, the proliferation of ECs, and the formation of functional capillary lumens. The onset of angiogenesis, called the angiogenic switch, begins by disrupting the local balance between pro-angiogenic and anti-angiogenic factors^15^. Frequently, tumor cells secrete pro-angiogenic molecules, including vascular endothelial growth factor (VEGF), fibroblast growth factor (FGF), platelet-derived growth factor (PDGF), epidermal growth factor (EGF), angiopoietin (Ang)-1, transforming growth factor (TGF), tumor necrosis factor (TNF), and MMPs, thereby leading to activation of their receptors on the surface of ECs^16^,^17^. In this regard, novel agents targeting vasculature in tumors are very distinct from conventional cytotoxic anti-cancer drugs and are considered to be promising approaches for the control of tumor growth and metastasis.

Recently, a number of angiogenesis inhibitors have been identified that have exhibited remarkable effects in the suppression of tumor growth and metastasis in experimental animal models. Among them, agents targeting VEGF signaling pathways, such as bevacizumab, sorafenib, and sunitinib, have been approved for cancer chemotherapy; many other angiogenesis inhibitors are in clinical trials^18^,^19^. However, these inhibitors have some limitations including high cost, undesirable side effects, and drug resistance^20^,^21^. Therefore, a search for anti-angiogenic agents synthesized from natural products is ongoing. Medicinal herbs, such as *Ocimum gratissimum*, *Patrinia scabiosaefolia*, and *Salvia officinalis*, have been shown to have anti-angiogenic activities^22^,^23^,^24^.

*Lophatheri Herba* (LH), known as “Dan Zhu Ye” in Chinese, the dried leaf of *Lophatherum gracile* Brongn (Poaceae), has long been used in Chinese medicine to treat fever and urinary tract inflammation and to relieve thirst. Pharmacological studies have demonstrated that extracts of LH show anti-bacterial, anti-pyretic, diuretic, and hyperglycemic properties^25^,^26^, and daily administration of the crude LH extract inhibits tumor growth^27^. Polysaccharides purified from LH possess strong anti-oxidant capabilities^28^, and oral administration of LH flavonoids exerts a protective effect on restraint stress-induced liver injury in mice^29^. In addition, flavone C-glycosides isolated from LH show potent anti-viral activity against human respiratory syncytial virus (RSV)^30^.

In the present study, we examined the influence of an ethanol extract of LH (ELH) on the metastatic and angiogenic properties of malignant tumor cells by using *in vitro* assays, an *in vivo* pulmonary metastasis model, *ex vivo* aortic ring assays, and *in ovo* chick chorioallantoic membrane (CAM) assays. Furthermore, we studied the underlying mechanisms of the anti-metastatic and anti-angiogenic activities of ELH and determined whether ELH suppresses tumor growth by using an *in ovo* xenograft model and whether it has the potential to control malignant tumors.

## Results

### ELH attenuates the metastatic potential of HT1080 cells at non-cytotoxic concentrations

**Figure 1:**
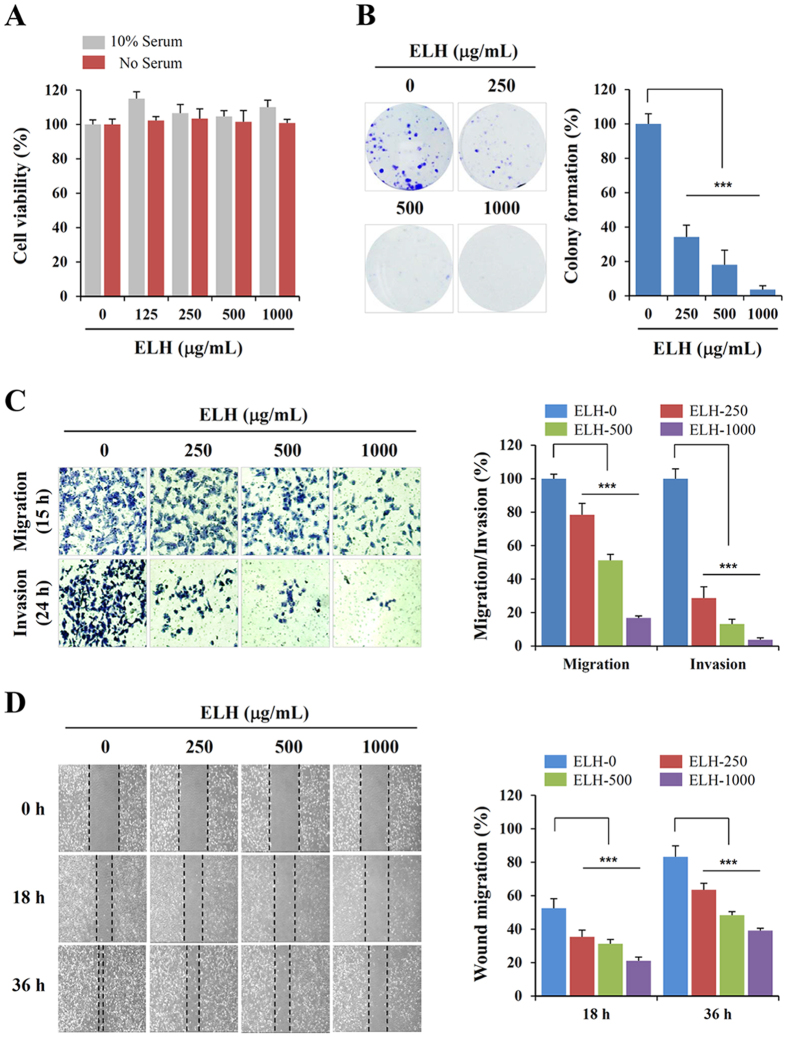
ELH decreases the metastatic potential of HT1080 cells. (**A**) HT1080 cells (5 × 10^3^ cells/well) seeded on 96-well culture plates were incubated with specified concentrations of ELH with or without serum for 48 h, and cell viability was evaluated by CCK assay. (**B**) Anchorage-dependent colony formation in the presence or absence of ELH was visualized by staining with crystal violet solution (n = 3 per group). (**C**) Cells pre-treated with or without ELH for 12 h were allowed to migrate across the Transwell and invade for 15 h and 24 h, respectively. Cells on the lower surface of the Transwell membrane were stained with crystal violet solution and observed under a phase contrast microscope (n = 5 per group). (**D**) After scratch wounds were made on the confluent cell monolayer, the effects of ELH on wound migration were monitored for 36 h. Wound migration was measured in five selected fields and calculated based on the width of injury at 0 h. Data are representative of three independent experiments and expressed as means ± SDs. Statistical significance was determined with Student’s *t*-test. ***p < 0.001 vs. untreated control.

To assess the *in vitro* anti-metastatic activity of ELH, we first assessed its cytotoxic activity in HT1080 cells by using a cell counting kit (CCK) assays. As shown in Fig. 1A, cell viability was not decreased by ELH treatment at concentrations up to 1000 μg/mL in the presence or absence of fetal bovine serum (FBS); thus, in this study, we treated cells with ELH at concentrations of 250, 500, and 1000 μg/mL. The ability of HT1080 cells to form sizable colonies from a single cell was significantly reduced by ELH treatment in a dose-dependent manner (F = 137.6, *p* < 0.0001, one-way ANOVA) (Fig. 1B); this effect was not due to cytotoxicity, as shown in Fig. 1A. In a Transwell cell culture system, serum-induced migration and invasion were decreased in a dose dependent manner in ELH-treated cells compared with control cells, showing reductions of approximately 85% and 95% at 1000 μg/mL, respectively (migration; F = 373.9, *p* < 0.0001, invasion; F = 421.6, *p* < 0.0001, one-way ANOVA) (Fig. 1C). In addition, control HT1080 cells rapidly migrated to the wound area, leading to approximately 52.5% and 83.2% healing at 18 and 36 h, respectively, based on the wound area at 0 h, whereas ELH treatment during incubation significantly suppressed wound migration in a dose-dependent manner by approximately 32.5–59.8% inhibition at 18 h and 23.6%–52.9% inhibition at 36 h compared with ELH-untreated control cells (18 h; F = 56.75, *p* < 0.0001, 36 h; F = 114.5, *p* < 0.0001, one-way ANOVA) (Fig. 1D). As demonstrated in the HT1080 cells, ELH treatment efficiently suppressed Transwell migration/invasion and wound migration of MDA-MB231 and DU145 cells (Supplementary Fig. S1).

### ELH reduces PMA-induced MMP expression and suppresses proteolytic activities in HT1080 cells

**Figure 2:**
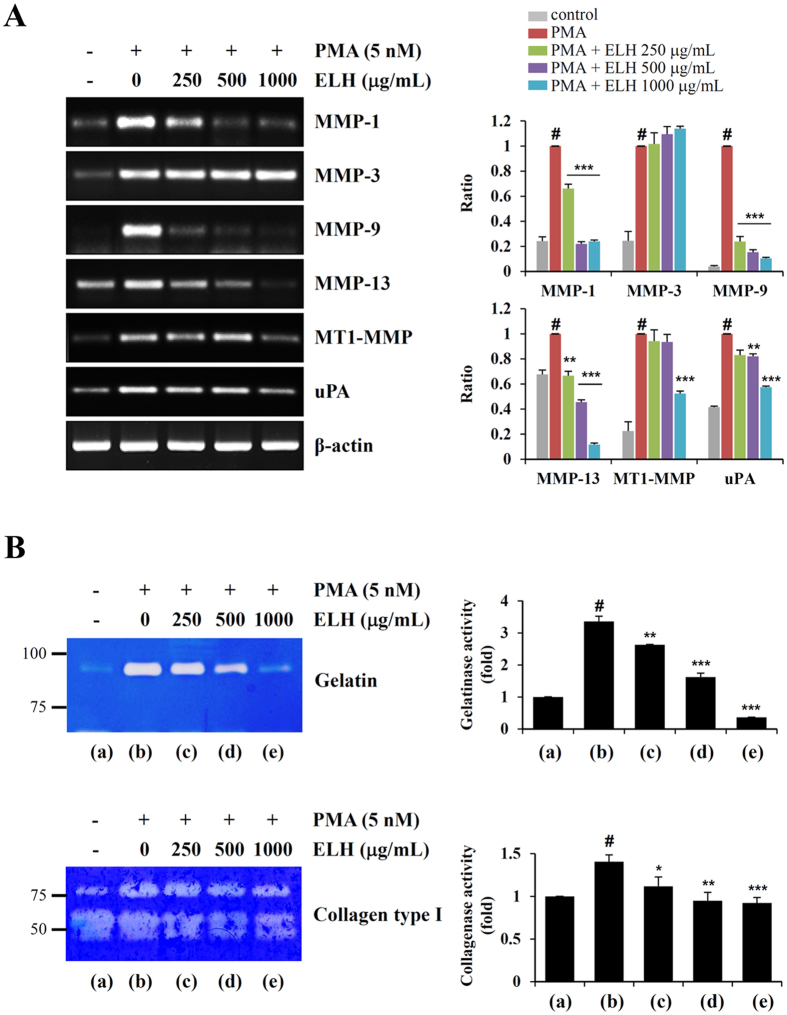
ELH inhibits PMA-induced elevation of MMP expression and the proteolytic activities of HT1080 cells. (**A**) Cells pre-treated with indicated concentrations of ELH for 12 h were stimulated with PMA (5 nM) under serum free conditions for an additional 24 h. The mRNA levels of MMPs were determined by RT-PCR using specific primers. Relative band intensities were calculated after normalization to β-actin expression. (**B**) Proteolytic activities in the collected conditioned medium (CM) were measured by zymography using gelatin and collagen type I as substrates. Band intensities were analyzed using ImageJ software. Data are representative of three independent experiments and expressed as means ± SDs (n = 3 per group). Statistical significance was evaluated with Student’s *t*-test. ^#^*p* < 0.001 vs. untreated control, *p < 0.05, **p < 0.01, and ***p < 0.001 vs. PMA stimulation.

To determine the anti-metastatic activity of ELH, we measured the transcriptional levels of MMPs including MMP-1, -3, -9, -13, MT1-MMP and urokinase-type plasminogen activator (uPA), in HT1080 cells by using semi-quantitative reverse transcription and polymerase chain reaction (RT-PCR). As shown in Fig. 2A, phorbol 12-myristate 13-acetate (PMA) stimulation markedly increased MMP expression, whereas ELH treatment efficiently inhibited the increase in the levels of MMP-1, -9, -13, MT1-MMP and uPA under PMA stimulation. In zymography analysis using gelatin and collagen type I as substrates, we found that ELH treatment significantly suppressed PMA-induced increases in the gelatinase and collagenase activities of HT1080 cells in a dose-dependent manner (gelatinase activity; F = 495.4, *p* < 0.0001, collagenase activity; F = 18.34, *p* = 0.0006, one-way ANOVA) (Fig. 2B).

### ELH blocks PMA-induced p38 and JNK phosphorylation as well as NF-κB activation in HT1080 cells

**Figure 3:**
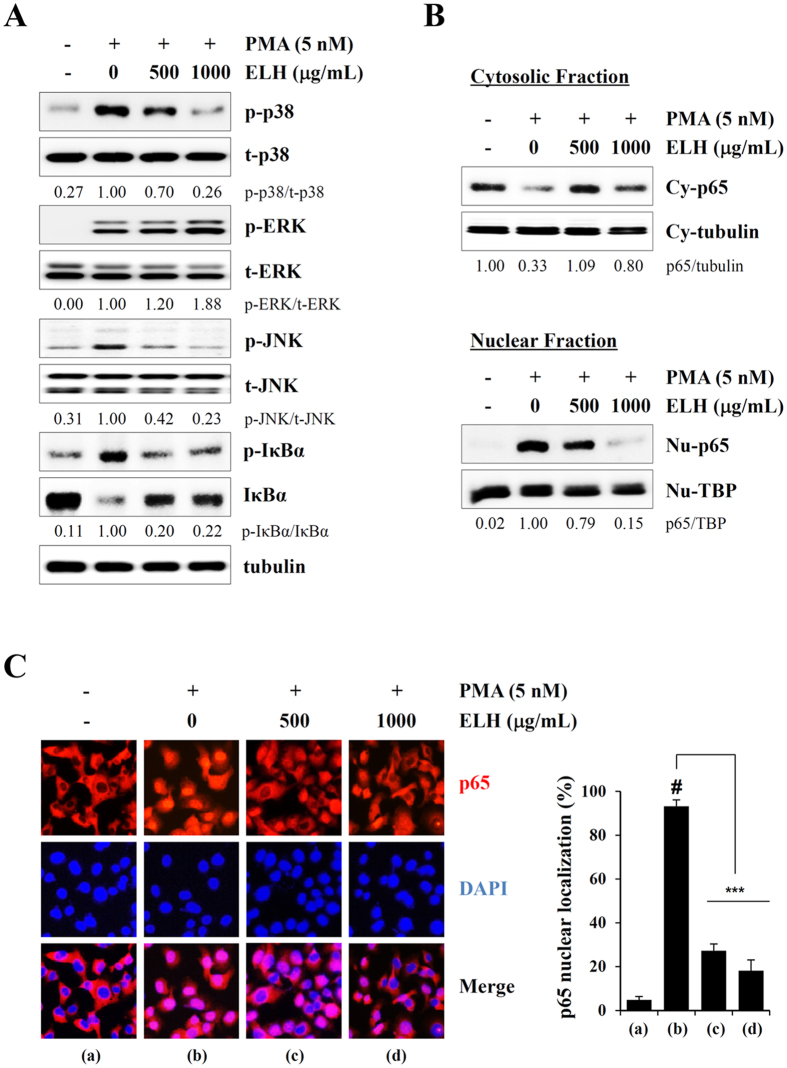
ELH suppresses PMA-induced p38, JNK, and NF-κB activation in HT1080 cells. (**A**) After treatment with 500 and 1000 μg/mL ELH for 12 h, cells were stimulated with PMA (5 nM) for 30 min. Total cell lysates were evaluated for MAPK phosphorylation and IκBα phosphorylation/degradation using western blotting. Band intensities were normalized to tubulin expression, and then the relative ratios of phosphorylated form/total form were calculated. The full size blot is shown in Supplementary Figure S2. (**B**) Cytosolic and nuclear compartments were isolated, and the effect of ELH on the nuclear translocation of the NF-κBp65 subunit under PMA stimulation was examined. Tubulin and TBP were used as loading controls for the cytosolic and nuclear fraction, respectively. (**C**) Cells were treated with 500 and 1000 μg/mL ELH for 12 h and then stimulated with 5 nM PMA for 30 min. Nuclear translocation of NF-κBp65 was monitored by an overlay of blue DAPI staining with red p65 immunofluorescence. Data are representative of three independent experiments and expressed as means ± SDs of five selected fields per sample. Statistical significance was evaluated with Student’s *t*-test. ^#^*p* < 0.001 vs. untreated control, ***p < 0.001 vs. PMA stimulation.

Because it has been reported that MAPKs and NF-κB activation play pivotal roles in the increase in MMP activity^31^,^32^, we next studied whether ELH downregulates these signaling pathways. In control HT1080 cells, PMA stimulation dramatically increased phosphorylation of p38, ERK, and JNK. In addition, IκBα phosphorylation accompanied by IκBα degradation was markedly increased by PMA stimulation. However, in ELH-treated HT1080 cells, PMA-induced p38 and JNK phosphorylation and increases in p-IκBα/IκBα ratio were almost completely blocked by ELH treatment (Fig. 3A). In addition, in control HT1080 cells, the NF-κBp65 subunit rapidly translocated from the cytosol to the nucleus after PMA stimulation, whereas in ELH-treated cells, PMA-induced NF-κBp65 nuclear translocation was significantly blocked, as demonstrated by western blotting (Fig. 3B) and immunocytochemistry (F = 597.9, *p* < 0.0001, one-way ANOVA) (Fig. 3C). These results collectively indicate that ELH possesses anti-metastatic potential via the suppression of proteolytic degradation of surrounding ECM via the blockade of p38, JNK, and NF-κB activation.

### ELH regulates production of angiogenesis-related proteins by suppressing hypoxia inducible factor (HIF)-1α accumulation under hypoxic conditions

**Figure 4:**
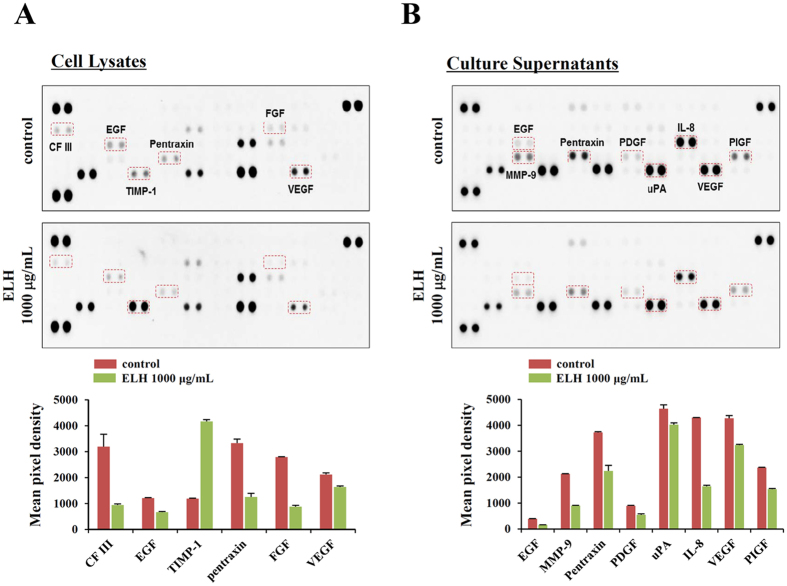
ELH reduces the levels of pro-angiogenic proteins in HT1080 cells. Cells were incubated with 1000 μg/mL ELH in complete medium for 24 h, washed with 0.5% FBS medium, and then further incubated for 24 h in 0.5% FBS medium under hypoxic conditions (1% O_2_). Levels of 55 angiogenesis-related proteins in cell lysates (**A**) and culture supernatants (**B**) were determined using a human angiogenesis array kit, and mean pixel densities were quantified using ImageJ software. Representative arrays from two independent experiments are shown, and data are expressed as means ± SDs calculated from duplicated dots.

**Figure 5:**
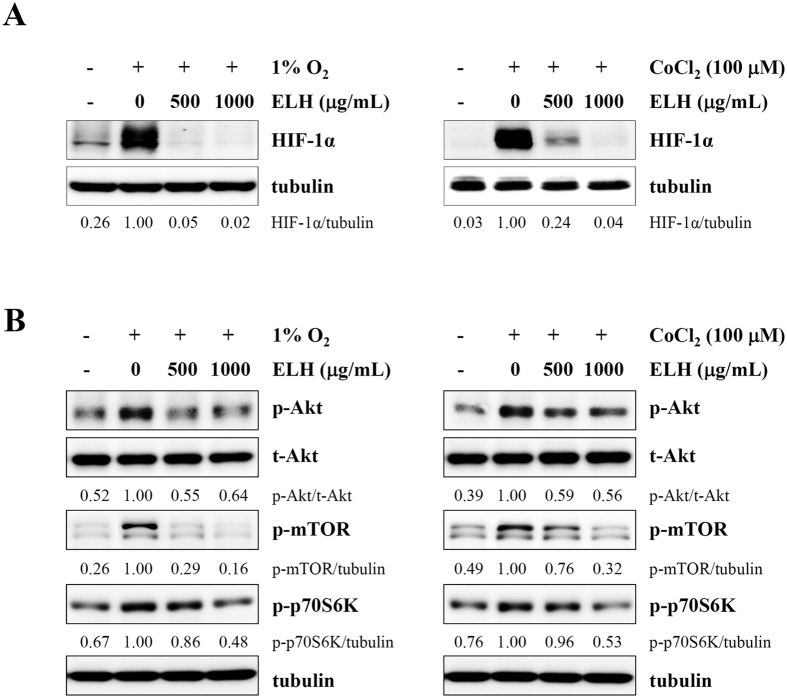
ELH inhibits hypoxia-induced accumulation of HIF-1α and phosphorylation of Akt, mTOR, and p70S6K in HT1080 cells. (**A,B**) Cells pre-treated with 500 and 1000 μg/mL ELH for 12 h were either incubated under hypoxia or stimulated with 100 μM CoCl_2_ for 6 h. The levels of HIF-1α and phosphorylated Akt, mTOR, and p70S6K in total cell lysates were determined using western blotting. Band intensities were analyzed using ImageJ software, and relative ratios were calculated after normalization to tubulin expression. Data are representative of two independent experiments. The full size blot is shown in Supplementary Figure S3.

Angiogenesis is necessary for tumor progression, invasion, and metastasis and depends on the release of pro-angiogenic factors by tumor cells and surrounding cells^14^. To assess the effects of ELH on tumor-induced angiogenesis, we analyzed cell lysates and culture supernatants obtained from control HT1080 cells and ELH-treated HT1080 cells under hypoxic conditions, using a Human Angiogenesis Proteome Profiler Array. As shown in Fig. 4A, the levels of pro-angiogenic proteins in cell lysates, including coagulation factor (CF) III, EGF, pentraxin, FGF, and VEGF, were significantly decreased by ELH treatment, whereas the levels of anti-angiogenic tissue inhibitor of metalloproteinase (TIMP)-1 were increased. Additionally, in culture supernatants, pro-angiogenic proteins, including EGF, interleukin (IL)-8, MMP-9, pentraxin, PDGF, placenta growth factor (PIGF), uPA, and VEGF, were significantly decreased in ELH-treated HT1080 cells compared with control HT1080 cells (Fig. 4B). Under hypoxic conditions, the HIF pathway acts as a key transcriptional regulator of tumor-induced angiogenesis^33^. Because pro-angiogenic factors, including VEGF, FGF, PDGF, MMP, and interleukins, are downstream targets of HIF-1α, we examined the effects of ELH treatment on HIF-1α accumulation and Akt/mTOR signaling under hypoxic conditions and CoCl_2_ treatment conditions, mimicking physiological hypoxia. As shown in Fig. 5, ELH treatment dramatically inhibited hypoxia- and CoCl_2_-induced HIF-1α accumulation, which was accompanied by suppression of Akt/mTOR/p70S6K phosphorylation, thus indicating that ELH decreased the production of pro-angiogenic factors via suppression of the HIF-1α pathway.

### ELH decreases tumor-induced angiogenesis in *in vitro*, *ex vivo*, and *in vivo* systems

**Figure 6:**
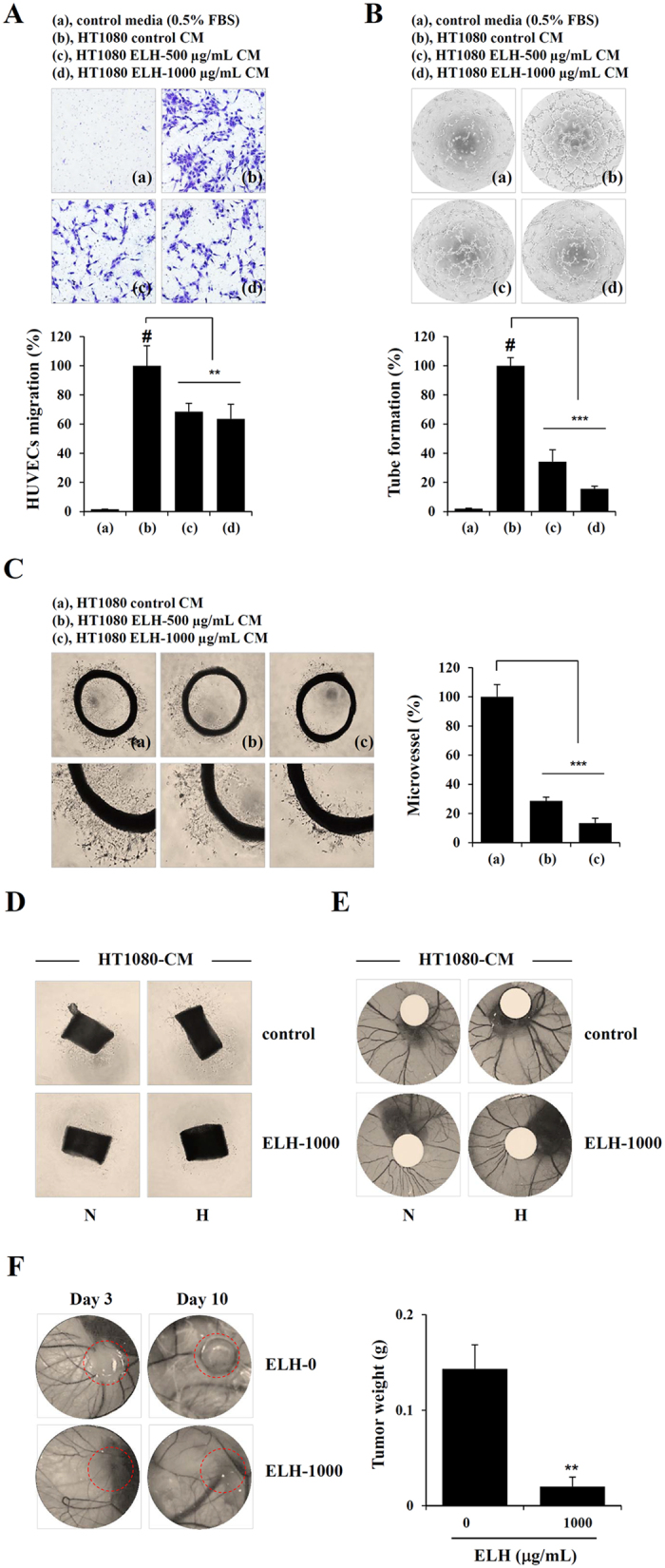
ELH suppresses tumor-induced angiogenesis and tumor growth. (**A**) ELH-treated or untreated HT1080 CM or 0.5% FBS medium (650 μL) were added to the lower chamber, and HUVECs (1 × 10^5^ cells) suspended in 0.5% FBS medium (100 μL) were loaded on the upper chamber of the Transwell, respectively. After incubation for 15 h, migrated HUVECs were observed under a phase contrast microscope after staining with crystal violet solution (n = 5 per group). Statistical significance was evaluated with Student’s *t*-test. ^#^*p* < 0.001 vs. 0.5% FBS medium, **p < 0.01 vs. control CM. (**B**) Capillary-like tube formation of HUVECs induced by ELH-treated or untreated CM of HT1080 cells was examined. The number of tubes in 3 different samples was counted. Statistical significance was evaluated with Student’s *t*-test. ^#^*p* < 0.001 vs. 0.5% FBS medium, ***p < 0.001 vs. control CM. (**C**) Rat aortic rings were placed on GFR-Matrigel-coated plates and incubated in EGM-2. On day 3, the EGM-2 was exchanged with ELH-treated or -untreated HT1080 CM and further incubated for 3 days. Aortic rings with sprouts were photographed, and the degree of sprouting was quantified using ImageJ software. Data are expressed as means ± SDs of five different samples. Statistical significance was evaluated with Student’s *t*-test. ***p < 0.001 vs. control CM. (**D**) Mouse aortic rings placed on GFR-Matrigel coated plates were incubated in EGM-2 for 3 days and then further incubated with ELH-treated or untreated HT1080 CM obtained from cells under normoxia (N) and hypoxia (H). After 5 days, aortic rings were photographed. (**E**) On ED 5, filter discs containing CM were carefully put on the CAM. After resealing the windows, the eggs were returned to the incubator and photographed at ED 10. (**F**) On ED 10, Matrigel plugs containing HT1080 cells and ELH were placed on the CAM and then further grown. After 10 days, tumor masses were photographed, excised from the CAM, and then weighed. Data are representative of three independent experiments and expressed as means ± SDs (n = 3 per group). Statistical significance was evaluated with Student’s *t*-test. **p < 0.01 vs. ELH-untreated control.

It has been reported that conditioned medium (CM) obtained from tumor cells induces angiogenesis of endothelial cells^34^,^35^. We therefore assessed the effects of ELH-treated or untreated CM from HT1080 cells on the angiogenic response. First, to determine the ability of endothelial cells to migrate across a Transwell, human umbilical vein endothelial cells (HUVECs) were seeded in the upper chambers, ELH-treated or untreated HT1080 CM filled in the lower chambers, and then HUVECs were allowed to migrate for 15 h. As shown in Fig. 6A, ELH-untreated HT1080 control CM strongly induced HUVECs migration, whereas ELH-treated HT1080 CM showed reduced migration of approximately 50~60% that of ELH-untreated HT1080 control CM, indicating that ELH suppressed tumor-induced migration of endothelial cells (F = 18.36, *p* < 0.0002, one-way ANOVA). Next, we examined the ability of HUVECs to form a tube-like network on Matrigel by incubation with ELH-treated or untreated HT1080 CM. As shown in Fig. 6B, CM from ELH-untreated HT1080 cells induced almost complete tube formation, whereas ELH-treated HT1080 CM did not cause robust tube formation in a dose-dependent manner (F = 173.8, *p* < 0.0001, one-way ANOVA). We also examined the effects of ELH-treated or untreated CM from HT1080 cells on microvessel sprouting by using a rat aortic ring assay, in which endothelial cell proliferation, migration, and tube formation occur simultaneously. ELH-untreated control CM from HT1080 cells efficiently induced microvessel sprouting around the aortic rings, whereas ELH-treated CM did not (Fig. 6C). Sprout length and the area of aortic rings incubated with ELH-treated CM were relatively shorter and smaller than those incubated with control CM, an effect that was dose-dependent (F = 216.5, *p* < 0.0001, one-way ANOVA), thus reinforcing the inhibitory effects of ELH on tumor-induced angiogenesis. To determine whether the inhibitory effect of ELH-treated CM was reversible, ELH-treated CM were removed, and the aortic rings were further incubated with Endothelial cell Growth Medium (EGM)-2. As shown in Supplementary Figure S4, incubation with EGM-2 for 3 days caused renewed vessel sprouting, thus indicating that inhibition of vessel sprouting by ELH-treated CM was not due to cytotoxicity. *Ex vivo* and *in vivo* angiogenesis was also tested using ELH-treated or untreated HT1080 CM obtained from normoxic and hypoxic conditions. Hypoxia enhanced the ability of HT1080 cells to induce mouse aortic ring sprouting, as well as chick chorioallantoic membrane (CAM) angiogenesis, compared with normoxia. However, ELH treatment suppressed tumor-mediated vessel formation and CAM angiogenesis under both normoxic and hypoxic conditions (Fig. 6D,E).

### ELH prevents *in ovo* tumor growth on a CAM and suppresses *in vivo* pulmonary metastasis of B16F10 cells with no toxic effects

Because CAMs are known to be immunodeficient hosts, grafted tissues or cells are able to survive on CAMs without species-specific restriction, and *in ovo* xenograft assays are useful for studying tumor growth. In previous studies, several osteosarcoma cell lines have been found to form solid tumors on CAMs, with a rich vascular response^36^,^37^. In this study, to assess the anti-cancer activity of ELH *in vivo*, we examined the ability of HT1080 cells to form solid tumors on CAMs with or without ELH treatment. As shown in Fig. 6F, control HT1080 cells rapidly proliferated and formed a sizable tumor mass, whereas ELH-treated HT1080 cells did not, thus resulting in an approximately 85% reduction in tumor weight. Next, to examine the *in vivo* anti-metastatic activity of ELH, we compared the pulmonary colonization of B16F10 cells injected intravenously via the tail vein between saline-treated and ELH-treated C57BL/6J mice. In ELH-administered mice, the number of colonies significantly decreased to approximately 35–55% of that in saline-administered control mice (F = 15.80, *p* = 0.0004, one-way ANOVA) (Supplementary Fig. S5). During the experimental period, there were no significant differences in body weight between control mice and ELH-administered mice (Supplementary Table S1). In agreement with the pulmonary colonization of B16F10 cells, the lung weights of ELH-treated mice were lower than those of saline-treated mice, while differences in other organ weights, including those of the liver, heart, spleen, and kidneys, were insignificant (Supplementary Table S2). To further confirm the safety of ELH, normal mice without tumors were treated daily with ELH at 50 and 100 mg/kg or the same volume of saline for 16 consecutive days. As shown in Supplementary Tables S3 and S4, ELH administration did not result in adverse effects in terms of loss of body weight and changes in organ weight. These data clearly indicate that ELH administration efficiently suppressed the pulmonary metastasis of B16F10 cells with no apparent side effects.

### Identification of the main components in ELH using HPLC

**Figure 7:**
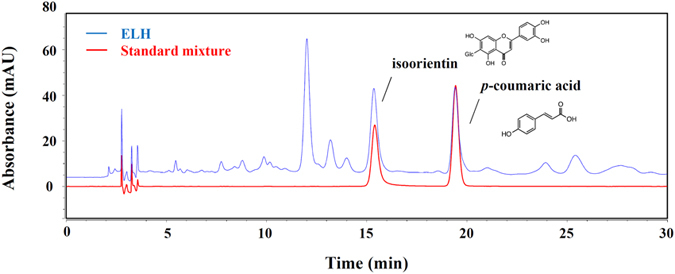
Chromatograms of major compounds in ELH. Two standard compounds, isoorientin (1) and *p*-coumaric acid (2), were detected in a standard mixture and in ELH using HPLC analysis at a wavelength of 331 nm.

Optimal separation was obtained using mobile phases consisting of 1% (v/v) aqueous acetic acid (solvent A) and 100% acetonitrite (solvent B). As shown in Fig. 7, two main components, including isoorientin and *p*-coumaric acid, in ELH were identified by comparing the retention time and UV spectra of standard samples. The retention times of isoorientin and *p*-coumaric acid were 15.36 min and 19.46 min, respectively. The major peak at the retention time between 11 and 13 min was not able to be identified; therefore, we are currently separating this peak for nuclear magnetic resonance (NMR) analysis.

## Discussion

Highly malignant tumors are capable of inducing angiogenesis to ensure a supply of oxygen and nutrients for survival. In addition to its role in tumor growth, angiogenesis offers a route for tumor cells to disseminate to distant organs via the blood stream and to then form metastases^11^,^14^. In the tumor microenvironment, pro-angiogenic and anti-angiogenic factors are produced by tumor and host cells, and these are strictly balanced. When the balance shifts in favor of pro-angiogenic factors, the angiogenic switch takes place, thus leading to the transition from a dormant to a hyper-vascularized tumor^12^,^13^,^16^,^17^. Hypoxia within the tumor microenvironment is thought to be a critical factor that up-regulates production of pro-angiogenic factors by tumor cells, leading to enhanced and rapid blood vessel formation^32^. In previous studies, hypoxic cells have been shown to be more aggressive and invasive, with potent metastatic ability^38^,^39^. The transcription factor HIF-1α subunit is involved in the differentiation of endothelial progenitor cells (EPCs) into ECs and in EC proliferation. Moreover, the HIF-1α subunit also induces the production of proteases, such as MMPs, thus facilitating sprouting and splitting of pre-existing vessels^40^,^41^. Hypoxia-induced epithelial-to-mesenchymal transition (EMT), characterized by activation of transcription factors, such as Snail and Slug, antagonizes p53-mediated apoptosis and promotes resistance to radiotherapy and chemotherapy with cisplatin and paclitaxel in ovarian cancer cells^42^,^43^. Therefore, targeting tumor-induced angiogenesis is widely considered to be a promising strategy that can limit the spread of cancer cells, improve the efficacy of cancer therapy and prolong the survival of cancer patients.

In the present study, we used CM obtained from HT1080 cells as stimulators of angiogenesis to mimic the tumor microenvironment. As reported previously^44^,^45^, CM from HT1080 cells dramatically promoted migration across Transwells and tube-like network formation on the Matrigel of HUVECs and increased the sprouting of microvessels from aortic rings; CM from ELH-treated HT1080 cells induced these changes to a lesser extent and caused no cytotoxicity (Fig. 6A–C). Under hypoxic conditions, ELH treatment almost completely inhibited hypoxia-induced HIF-1α accumulation and suppressed Akt/mTOR/p70S6K activation (Fig. 5), thus resulting in downregulation of target genes, including angiogenic factors and MMPs (Figs 2 and 4). Hypoxia-induced sprouting of microvessels and angiogenesis in the CAM was significantly decreased by ELH treatment (Fig. 6D,E). *In ovo* xenograft assays revealed that ELH-treated HT1080 cells did not grow on the CAM, whereas ELH-untreated control HT1080 cells formed a measurable tumor mass with high vasculature (Fig. 6F), thus indicating that ELH dramatically decreased tumor growth by inhibiting angiogenesis rather than by inducing cytotoxicity. In previous studies, the ethanol extract of *Patrinia scabiosaefolia* (EEPS) has been shown to inhibit colorectal cancer (CRC) growth via suppression of tumor angiogenesis, including reduction of VEGF secretion by tumors and inhibition of the proliferation, migration, and tube formation of HUVECs^23^. In addition, extracts of *Cordyceps militaris* and *Trametes robiniophila* Murr. (Huaier) show potent anti-tumor activity against human melanoma and murine breast cancer, respectively, through the induction of apoptosis and suppression of the angiogenic potential of HUVECs, thus strongly supporting the key role of angiogenesis in the progression of tumors^46^,^47^. In addition to its anti-angiogenic activity, ELH significantly suppressed cellular metastatic activities, including migration, invasion, and proteolysis, via suppression of p38, JNK and NF-κB activation (Figs 1, 2, 3). Moreover, in an *in vivo* pulmonary metastasis experiment, oral ELH administration suppressed metastatic colonization of B16F10 melanoma cells in the lungs of C57BL/6J mice and caused no apparent toxic effects, collectively suggesting that ELH can be used as a treatment for malignant cancer (Fig. S5 and Tables S1–4).

Among the compounds identified in ELH, *p*-coumaric acid has been shown to possess potent anti-cancer activities by inhibiting angiogenesis *in vivo* and to inhibit the growth of HCT-15 colon cancer cells by inducing apoptosis through ROS-mediated mitochondrial dysfunction^48^,^49^. In addition, isoorientin has been shown to inhibit TNF-α-mediated production of IL-6, IL-8, and VEGF in human HaCaT cells and to suppress cell migration in 3T3 murine fibroblasts^50^. Moreover, isoorientin has been reported to induce apoptosis in HepG2 cells through mitochondrial damage by inhibiting PI3K/Akt/ERK signaling and activating p38/JNK signaling^51^. These results suggest that these compounds in ELH may contribute to its anti-cancer properties, including suppression of metastasis, angiogenesis, and tumor growth in HT1080 cells.

In summary, we demonstrated that ELH suppresses the metastatic and angiogenic potential of highly malignant HT1080 cells through decreasing MMPs and pro-angiogenic factors. Furthermore, we observed the therapeutic efficacy of ELH against tumor growth in *in ovo* xenografts and pulmonary metastasis in mice, thus indicating that ELH may be a safe and potent anti-cancer agent for treating malignant human cancers.

## Materials and Methods

### Cell culture

Human fibrosarcoma HT1080 cells (KCLB No. 10121), human breast adenocarcinoma MDA-MB231 (KCLB No. 30026), human prostate carcinoma DU145 (KCLB No. 30081), and murine melanoma B16F10 cells (KCLB No. 80008) were purchased from Korean Cell Line Bank (Seoul, Korea). Cells were grown in Roswell Park Memorial Institute (RPMI) 1640 or Dulbecco’s modified Eagle’s medium (DMEM; Lonza, Walkersville, MD, USA) supplemented with 10% FBS (Cellgro, Manassas, VA, USA) and antibiotics (100 U/ml penicillin/100 μg/ml streptomycin; Cellgro) at 37 °C in a humidified 5% CO_2_ incubator. HUVECs obtained from Innopharmascreen (Asan, Korea) were maintained in EGM-2 (PromoCell, Heidelberg, Germany) and used for experiments at passages 3 to 8.

### Animals

Five-week-old female C57BL/6J mice and ICR mice were purchased from Orientbio (Sungnam, Korea). Male Sprague-Dawley rats were purchased from Taconic Farms Inc. (Samtako Bio Korea, Osan, Korea). Mice and rats were housed in a specific-pathogen-free facility under controlled conditions (12 h-light/12 h-dark cycles at 22 ± 1 °C and 55 ± 5% humidity). All animal experiments with reference numbers #14‒054, #15–085, and #16–003 were approved by the Animal Care and Use Committee of the Korea Institute of Oriental Medicine (KIOM, Daejeon, Korea) and were performed in accordance with their guidelines.

### Reagents and antibodies

Type I collagen from calf skin, type A gelatin from porcine skin, PMA, and mitomycin C from *Streptomyces caespitosus* were purchased from Sigma Chemical Co. (St. Louis, MO, USA). Growth factor-reduced Matrigel basement membrane matrix was obtained from BD Biosciences (Bedford, MA, USA). Antibodies against p38, p-p38, ERK, p-ERK, JNK, p-JNK, IκBα, p-IκBα, p65, Akt, p-Akt, p-mTOR, p-p70S6K, TBP, and tubulin were purchased from Cell Signaling Technology (Danvers, MA, USA). Anti-HIF-1α antibody was obtained from BD Biosciences, and horseradish peroxidase-conjugated anti-mouse and anti-rabbit antibodies were obtained from Cell Signaling Technology.

### Preparation of ELH

Dried *L. Herba* purchased from Yeongcheon Oriental Herbal Market (Yeongcheon, Korea) was certified for its identity by Professor Ki Hwan Bae (College of Pharmacy, Chungnam National University, Daejeon, Korea) and then stored at the herbal bank of KIOM. To prepare ELH, dried *L. Herba* (30 g) was ground into a fine powder, soaked in 300 mL of 70% ethanol, and extracted in a shaking incubator at 40 °C for 24 h. The extract was filtered through a testing sieve (150 μm, Retsch, Haan, Germany), evaporated on a rotary evaporator, concentrated by lyophilization, and then stored at −20 °C. For *in vitro* experiments, ELH powder (50 mg) was dissolved in 1 mL of 10% DMSO (v/v) and filtered through a 0.22 μm disk filter.

### Cell cytotoxicity and colony formation assay

To examine the effect of ELH on proliferation, cells plated in 96-well plates (5 × 10^3^ cells/well) were treated with the indicated concentrations of ELH for 48 h and then assessed for cell viability using a CCK-8 (Dojindo Laboratories, Kumamoto, Japan), according to the manufacturer’s instructions. To assess the ability of anchorage-dependent colony formation, cells seeded in 12-well culture plates (5 × 10^2^ cells/well) were incubated in the presence or absence of ELH for 7 days, and then colonies were stained with 0.2% crystal violet/20% methanol (w/v) solution for 30 min, washed with distilled water, and photographed.

### Cell migration and invasion assay

The ability of cells to migrate to the wounded area was assessed as described previously^52^. Briefly, 90% confluent cells on 60 mm culture dishes were treated with mitomycin C (25 μg/mL) for 30 min and wounded by scraping. After floating cell debris was washed away, complete culture medium was added, and cell migration in the presence or absence of ELH was monitored. Cell migration and invasion across a Transwell chamber with a 6.5 mm diameter and an 8 μm pore polyethylene tetraphthalate (PET) membrane (SPL Lifesciences, Korea) were measured as described previously^52^.

### Zymography

The activity of MMP secreted into culture medium was measured by zymography using gelatin and collagen as substrates, as described previously^52^. The activity of gelatinase at 92 kDa and collagenase at 50‒75 kDa was detected as clear bands with blue backgrounds.

### Reverse transcription and polymerase chain reaction (RT-PCR)

Total RNA was extracted using an RNA extraction solution (BioAssay Co., Daejeon, Korea) and reverse transcribed to cDNA using a 1^st^ Strand cDNA synthesis kit (BioAssay Co.), according to the manufacturer’s protocol. cDNA aliquots were amplified by PCR using specific primers. PCR products were electrophoresed on 1% agarose gels and visualized by GreenLight (BioAssay Co.).

### Immunoblot analysis

Total cell lysates and nuclear/cytosolic fractions were obtained using M-PER Mammalian Protein Extraction Reagent and NE-PER Nuclear and Cytosolic Extraction Reagent (Thermo Scientific, Rockford, IL), respectively. After determining protein concentrations using a Bicinchoninic Acid (BCA) kit (Sigma), protein aliquots (20 μg) were resolved by SDS-PAGE and immunoblotted with specific antibodies, as described previously^52^. Proteins were visualized with a Bio-Rad Clarity Western ECL Substrate and ChemiDoc Touch Imaging System (Bio-Rad, Hercules, CA).

### Immunocytochemistry for NF-κBp65 nuclear translocation

Cells grown in 35 mm cover glass bottom dishes (SPL Lifesciences) were treated with ELH for 12 h and then stimulated with 5 nM PMA for 30 min. After being washed with cold PBS three times, the cells were fixed with 4% paraformaldehyde (PFA) in PBS for 30 min at RT, permeabilized and blocked with ABS buffer (1 M Tris Base, 1.5 M NaCl, pH 7.5) containing 0.1% Triton X-100 and 3% goat serum for 30 min at RT. Localization of NF-κBp65 was detected with mouse anti-p65 antibody (diluted 1:1000 in ABS buffer) O/N at 4 °C, and this was followed by Alexa 568-goat anti-mouse IgG antibody (1:1000) for 3 h at RT. After nuclear counterstaining with DAPI, cells were analyzed under a fluorescence microscope (Nikon Eclipse Ti).

### Proteome profiler antibody arrays

The expression profiles of 55 proteins involved in angiogenesis and invasiveness in culture supernatants and lysates were analyzed using a Proteome Profiler Human Angiogenesis Array kit (R&D Systems, Minneapolis, MN, USA), according to the manufacturer’s instructions.

### Capillary-like tube formation assay

The ability of HUVECs to form tubular structures on a basement membrane was evaluated using a Cultrex *in vitro* angiogenesis assay kit (Trevigen, Gaithersburg, MD, USA), according to the manufacturer’s protocol. In brief, ice-chilled basement membrane extract (BME, 50 μL) was evenly distributed across each well of a 96-well plate, and the plate was incubated at 37 °C for 30 minutes to solidify. HUVECs (5 × 10^4^ cells/well/100 μL) suspended in ELH-treated CM were added into each well containing solidified BME and incubated for 4 to 12 h at 37 °C in a CO_2_ incubator. Tube-like networks were visualized through phase contrast inverted light microscopy. For the preparation of CM, cells were incubated for 24 h in complete medium containing ELH, washed twice with 0.5% FBS medium, and then further incubated for 24 h in 0.5% FBS medium. After harvesting, CM were centrifuged at 12,000 rpm for 10 min to remove cell debris and stored at −80 °C until use.

### *Ex vivo* angiogenesis assay

Dorsal thoracic aortas were obtained from rats under aseptic conditions and rinsed in cold PBS. After removal of the surrounding connective tissue, the aortas were evenly cut into rings of 1 mm thickness using a surgical blade. Each ring was placed on pre-coated Matrigel (100 μL) in 48-well culture plates and covered with 40 μL of Matrigel. After polymerization, the rings were incubated for 3 days after adding 400 μL of EGM-2. After verifying sprouts from the aortic rings, the EGM-2 was replaced with ELH-untreated control CM and ELH-treated CM, and microvessel outgrowths were checked daily under phase contrast inverted light microscopy and photographed. Aortic rings isolated from ICR mice were also used to perform the angiogenesis assay.

### Chick embryo chorioallantoic membrane (CAM) assay

Fertilized white Leghorn chicken eggs were obtained from Deogi Farm (Yongin, Korea) and incubated in an egg incubator (R-COM, Gimhae, Korea) at 37 °C with 70% humidity. We designated this time point as chick embryonic development day (ED) 0. To examine the anti-proliferative efficacy of ELH, initially, a small window was created in the shell on ED 3 after albumin removal and resealed with adhesive tape, and then the egg was returned to the incubator. On ED 10, cells (1 × 10^6^ cells) mixed with cold growth factor-reduced (GFR) Matrigel (100 μL) and solidified at 37 °C for 30 min were placed on the top of the CAM and returned to the incubator after resealing the windows. After 10 days, the tumor graft was harvested from each embryo and weighed. To examine the anti-angiogenic activity of ELH, each CM (20 μL) was loaded on the sterilized 5 mm filter disc and then applied to the CAM on ED 5. After 5 days, the vasculature was photographed.

### *In vivo* pulmonary metastasis experiment

To initiate pulmonary metastasis, B16F10 cells (2 × 10^5^ cells) suspended in 200 μL of PBS were injected into 5-week-old female C57BL/6J mice via the tail vein. After being randomly divided into groups, the mice (n = 5 per group) were administered vehicle (saline) or ELH daily at 50 and 100 mg/kg, and body weights were measured every two days during the experiment. On day 21, the mice were sacrificed, their lungs were weighed, and the metastatic black colonies were macroscopically counted.

### High performance liquid chromatography (HPLC) analysis

Chromatographic analysis of ELH was performed with a Hitachi HPLC system, and data were processed using EZchrom Elite software (Lachrom Elite; Hitachi High-Technologies Co., Tokyo, Japan). Separation was performed with a Phenomenex Luna C_18_ column (Part No. 00G-4252-E0, 5 μm particle size, 100 Å, 4.6 × 250 mm, Phenomenex Co., Torrance, CA, USA) at 25 °C, and the injection volume was 3 μL. Gradient elution was performed using solvent A (1% aqueous acetic acid, v/v) and solvent B (100% acetonitrite); the gradient flow was as follows: 0–15 min with 14.5% B, 15–35 min with 14.6% B, 35–45 min with 100% B, and 45–60 min with 14.5% B. The flow rate was 1 mL/min, and HPLC chromatograms were obtained using a UV detector at 190–400 nm. Standard samples including isoorientin and *p*-coumaric acid (Sigma) were dissolved in methanol at 100 ppm, and the ELH sample was dissolved in methanol at 20 mg/mL.

### Statistics

Data were expressed as means ± standard deviation (SD). Statistical significance of mean values in two groups was analyzed by Student’s *t*-test. Treatment effect was analyzed with one-way ANOVA by Dunnett’s test. All variables were analyzed using GraphPad PRISM software (GraphPad PRISM software Inc., Version 6.07, CA, USA). A *p*-value less than 0.05 was considered as statistically significant.

## Additional Information

**How to cite this article**: Kim, A. *et al*. Ethanol extract of *Lophatheri Herba* exhibits anti-cancer activity in human cancer cells by suppression of metastatic and angiogenic potential. *Sci. Rep*. **6**, 36277; doi: 10.1038/srep36277 (2016).

**Publisher’s note:** Springer Nature remains neutral with regard to jurisdictional claims in published maps and institutional affiliations.

## Supplementary Material

Supplementary Information
