# Supplementary material for: Ethanol extract of Lophatheri Herba exhibits anti-cancer activity in human cancer cells by suppression of metastatic and angiogenic potential
Source: Sci Rep. 2016 Nov 3;6:36277. doi: 10.1038/srep36277 (PMC5093764; doi:10.1038/srep36277)
Supplement: Supplementary Information [file srep36277-s1.pdf]

Supplementary Information

Ethanol extract of *Lophatheri Herba* exhibits anti-cancer activity in human cancer cells by suppression of metastatic and angiogenic potential

Aeyung Kim, Minju Im, Min Jung Gu and Jin Yeul Ma*

- Supplementary Figure S1
- Supplementary Figure S2
- Supplementary Figure S3
- Supplementary Figure S4
- Supplementary Figure S5
- Supplementary Table S1
- Supplementary Table S2
- Supplementary Table S3
- Supplementary Table S4

*Correspondence and requests for materials should be addressed to J.Y.M (jyma@kiom.re.kr)

Figure S1

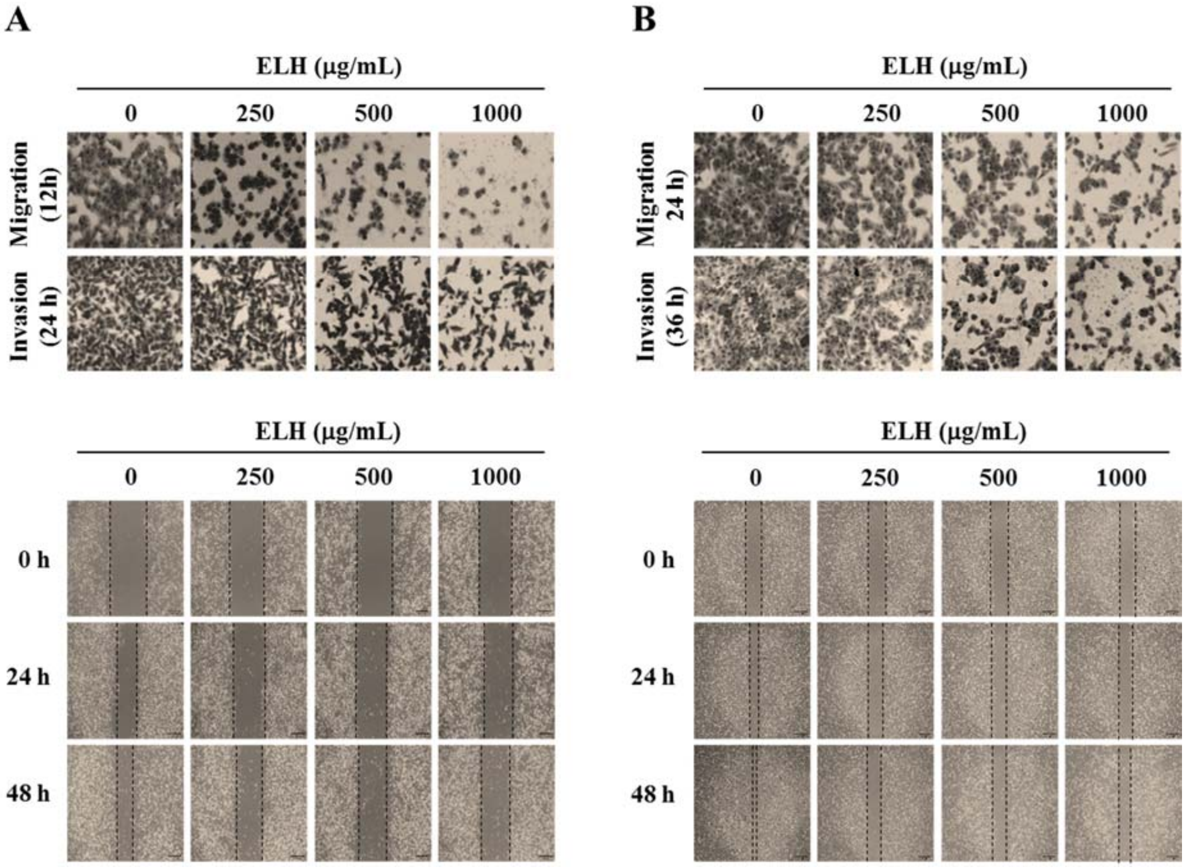

Figure S1. ELH suppresses the metastatic potential of MDA-MB231 (A) and DU145 (B) cells. Cells were pretreated with indicated concentrations of ELH for 12 h, and then subjected to determine the ability to migrate and invade across Transwell (upper) as well as migrate to wound area (lower) at indicated time points.

Figure S2

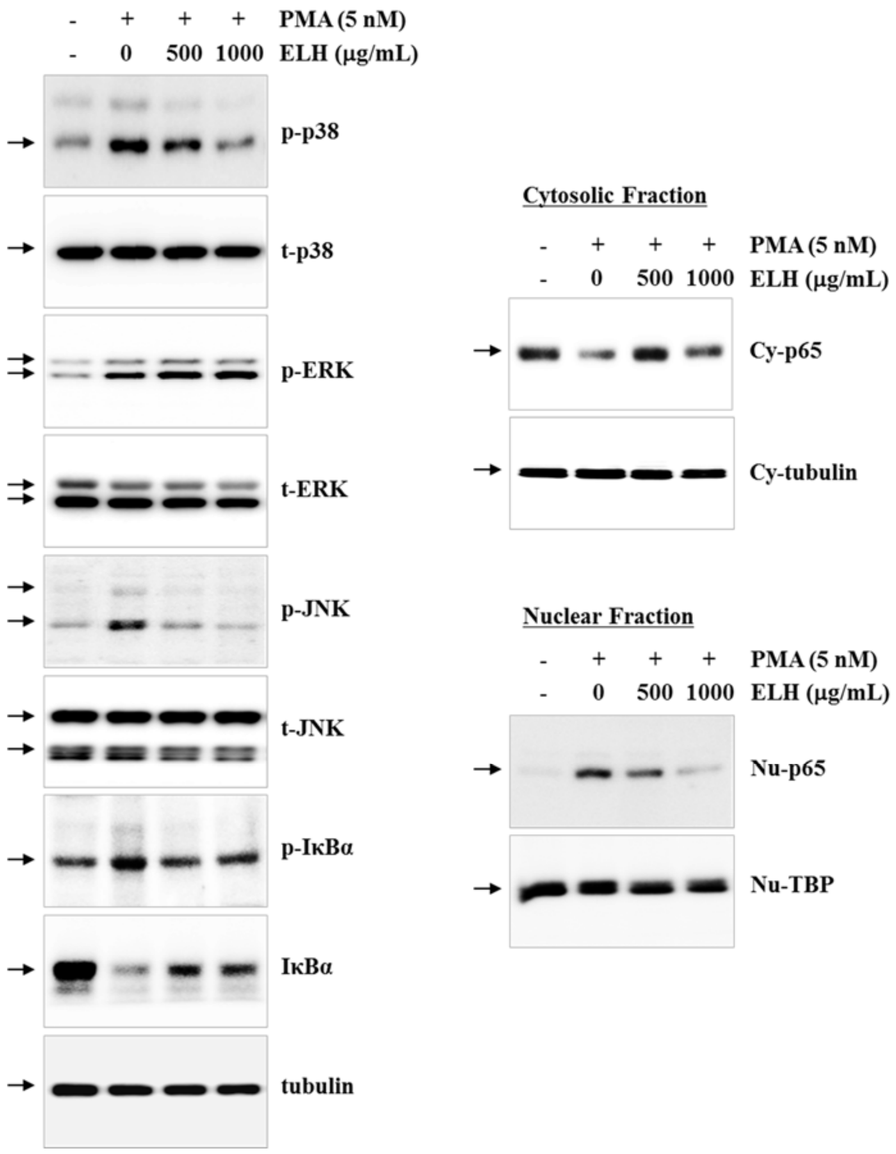

Figure S2. ELH suppresses PMA-induced p38 and JNK phosphorylation as well as NF-κB activation in HT1080 cells. This is a full length image of the cropped blots presented in the Figure 3A and 3B.

Figure S3

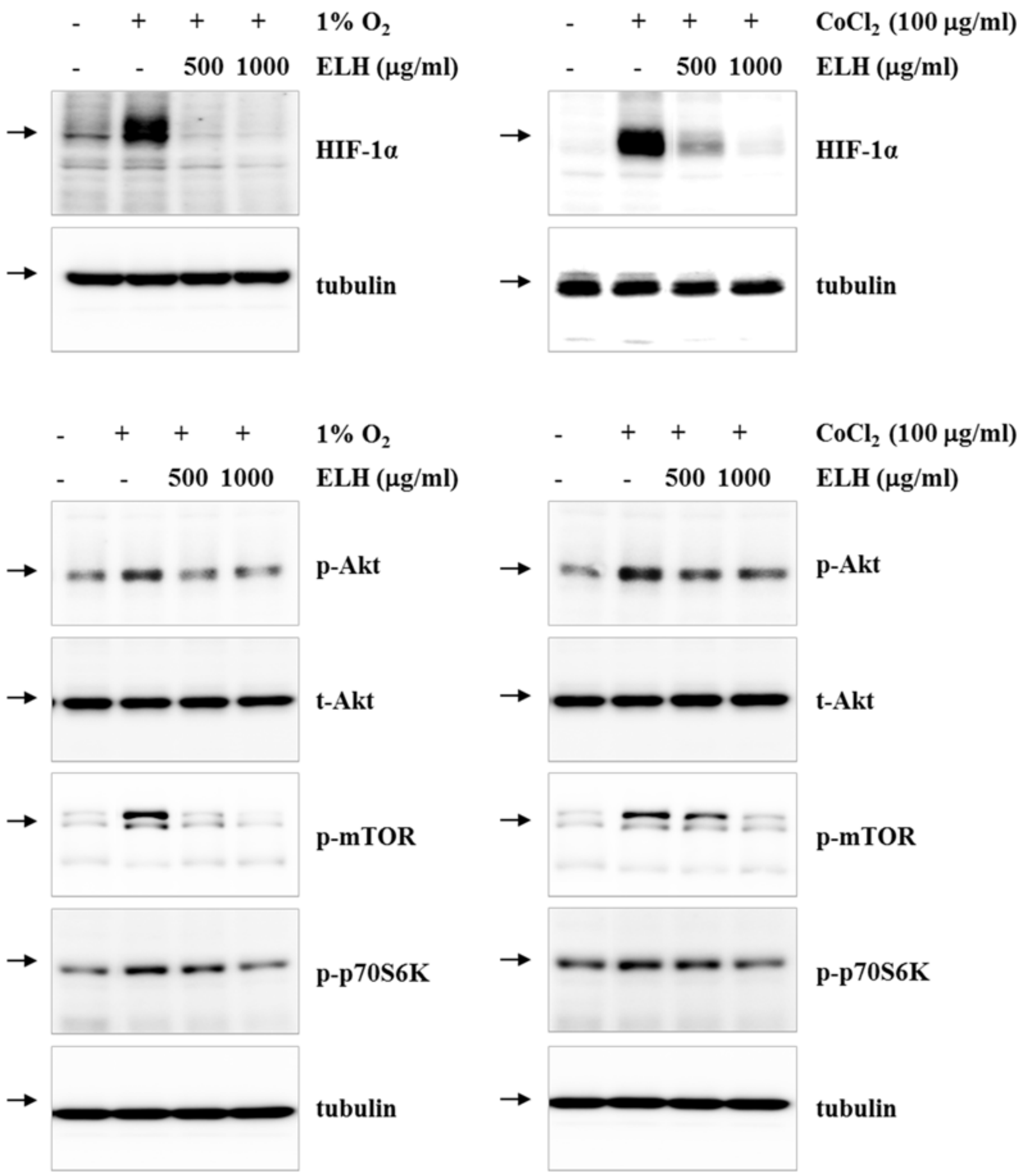

Figure S3. ELH inhibits hypoxia-induced HIF-1α accumulation and Akt/mTOR/p70S6K phosphorylation in HT1080 cells. This is a full length image of the cropped blots presented in the Figure 5A and 5B.

Figure S4

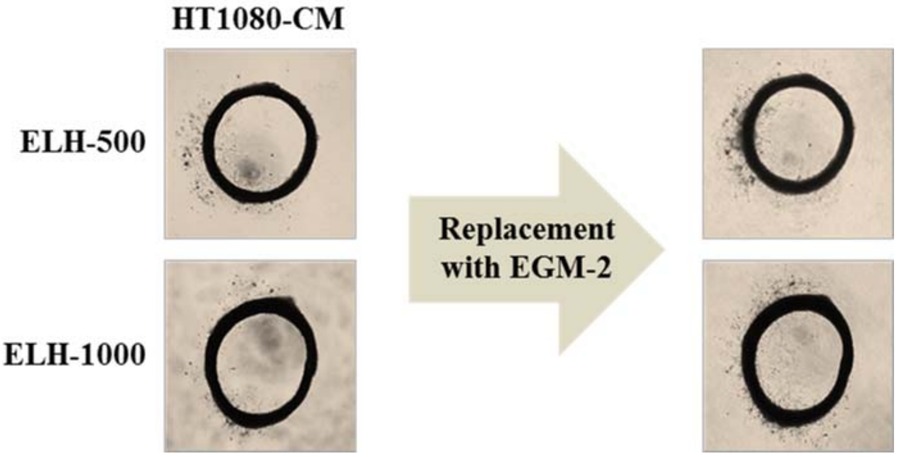

Figure S4. Inhibitory effect of ELH on vessel sprout formation is not due to the cytotoxicity. Aortic rings incubated with ELH-treated HT1080 CMs were further incubated in EGM-2 media for additional 3 days after CMs were replaced.

Figure S5

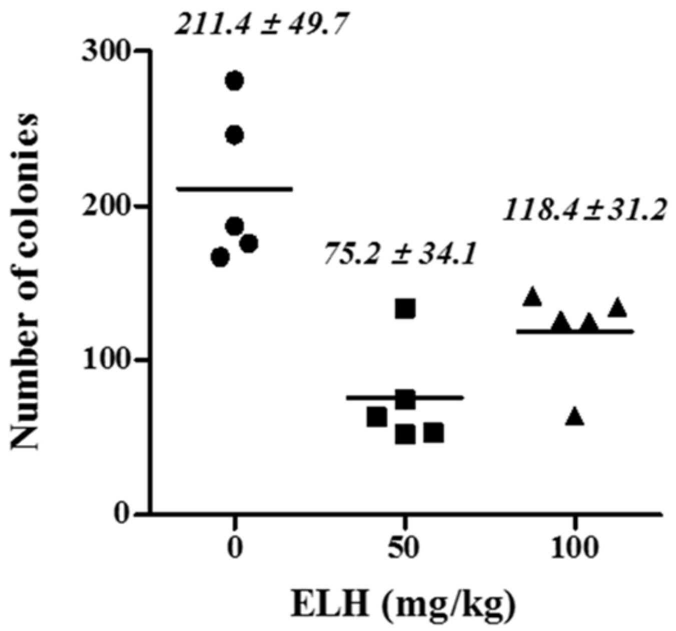

Figure S5. ELH inhibits the *in vivo* pulmonary metastasis. B16F10 cells (2 × 10^5/mouse in 200 μL PBS) were injected into the tail vein of C57BL/6J mice and then orally administered with 50 and 100 mg/kg ELH (n=5 per group). On day 21, mice were sacrificed and colonies on the lung surface were counted macroscopically. Data are presented as means ± S.D. Statistical significance was evaluated with Student *t*-test. $^{**}p < 0.01$, $^{***}p < 0.001$ vs. saline-treated mice.

Table S1. Body weight of mice administered with ELH or saline

Treatment	Body weight (g)				
	Day 0	Day 8	Day 12	Day 16	Day 20
Saline	18.84 ± 0.64	19.22 ± 0.44	19.76 ± 0.37	20.24 ± 0.48	20.53 ± 1.31
50 mg/kg	18.83 ± 0.87	19.41 ± 0.41	19.93 ± 0.72	20.02 ± 0.74	20.12 ± 0.86
100 mg/kg	19.32 ± 0.68	19.77 ± 0.70	20.21 ± 0.74	20.53± 0.92	20.31 ± 1.09

Each group of female C57BL/6J mice (n=5) were intravenously injected with B16F10 cells (3×10^5 cells/mice) and then orally administered with 50 and 100 mg/kg ELH or same volume of saline daily for 20 days. Body weights were weighed at 0, 4, 8, 12, 16, and 20 days. Data are expressed as mean ± S.D.

Table S2. Organ weight of mice administered with ELH or saline

Treatment	Organ weight (g)					
	Lung	Liver	Heart	Spleen	Kidney (L)	Kidney (R)
Saline	0.51 ± 0.08	0.94 ± 0.10	0.10 ± 0.01	0.09 ± 0.02	0.12 ± 0.01	0.12 ± 0.01
50 mg/kg	0.25 ± 0.03***	1.02 ± 0.12	0.09 ± 0.01	0.09 ± 0.01	0.13 ± 0.01	0.12 ± 0.01
100 mg/kg	0.25 ± 0.05***	1.01 ± 0.08	0.10 ± 0.01	0.09 ± 0.01	0.13 ± 0.01	0.12 ± 0.01

Each group of female C57BL/6J mice (n=5) were intravenously injected with B16F10 cells (3×10^5 cells/mice) and then orally administered with 50 and 100 mg/kg ELH or same volume of saline daily for 20 days. At day 20, mice were sacrificed and organs were weighed. Data are expressed as mean ± S.D. Statistical significance was evaluated with Student *t*-test. ***$p < 0.001$ vs. saline-treated mice.

Table S3. Body weight of mice administered with ELH or saline

Treatment	Body weight (g)		
	**Day 0**	**Day 8**	**Day 16**
Saline	18.58 ± 0.63	19.29 ± 0.60	21.32 ± 0.73
50 mg/kg	18.37 ± 0.19	18.90 ± 0.29	20.80 ± 0.30
100 mg/kg	18.82 ± 0.41	19.73 ± 0.34	21.47 ± 0.64

Each group of female C57BL/6J mice (n=3) were orally administered with 50 and 100 mg/kg ELH or same volume of saline daily and weighed body weight at 0, 4, 8, 12, and 16 days. Data are expressed as mean ± S.D.

Table S4. Organ weight of mice administered with ELH or saline

Treatment	Organ weight (g)					
	Lung	Liver	Heart	Spleen	Kidney (L)	Kidney (R)
Saline	0.15 ± 0.02	1.18 ± 0.01	0.10 ± 0.01	0.09 ± 0.01	0.14 ± 0.01	0.13 ± 0.01
50 mg/kg	0.14 ± 0.01	1.17 ± 0.01	0.10 ± 0.00	0.09 ± 0.01	0.13 ± 0.01	0.13 ± 0.00
100 mg/kg	0.17 ± 0.01	1.18 ± 0.10	0.11 ± 0.01	0.09 ± 0.00	0.14 ± 0.01	0.13 ± 0.01

Each group of female C57BL/6J mice (n=3) were orally administered with 50 and 100 mg/kg ELH or same volume of saline daily. At day 16, mice were sacrificed and organs were weighed. Data are expressed as mean ± S.D.
